# Predicting Future Morphological Changes of Lesions from Radiotracer Uptake in 18F-FDG-PET Images

**DOI:** 10.1371/journal.pone.0057105

**Published:** 2013-02-19

**Authors:** Ulas Bagci, Jianhua Yao, Kirsten Miller-Jaster, Xinjian Chen, Daniel J. Mollura

**Affiliations:** 1 Center for Infectious Disease Imaging (CIDI), National Institutes of Health, Bethesda, Maryland, United States of America; 2 Radiology and Imaging Sciences, Clinical Center, National Institutes of Health, Bethesda, Maryland, United States of America; 3 School of Electronics and Information Engineering, Soochow University, Suzhou City, China; The University of Chicago, United States of America

## Abstract

We introduce a novel computational framework to enable automated identification of texture and shape features of lesions on ^18^F-FDG-PET images through a graph-based image segmentation method. The proposed framework predicts future morphological changes of lesions with high accuracy. The presented methodology has several benefits over conventional qualitative and semi-quantitative methods, due to its fully quantitative nature and high accuracy in each step of (i) detection, (ii) segmentation, and (iii) feature extraction. To evaluate our proposed computational framework, thirty patients received 2 ^18^F-FDG-PET scans (60 scans total), at two different time points. Metastatic papillary renal cell carcinoma, cerebellar hemongioblastoma, non-small cell lung cancer, neurofibroma, lymphomatoid granulomatosis, lung neoplasm, neuroendocrine tumor, soft tissue thoracic mass, nonnecrotizing granulomatous inflammation, renal cell carcinoma with papillary and cystic features, diffuse large B-cell lymphoma, metastatic alveolar soft part sarcoma, and small cell lung cancer were included in this analysis. The radiotracer accumulation in patients' scans was automatically detected and segmented by the proposed segmentation algorithm. Delineated regions were used to extract shape and textural features, with the proposed adaptive feature extraction framework, as well as standardized uptake values (SUV) of uptake regions, to conduct a broad quantitative analysis. Evaluation of segmentation results indicates that our proposed segmentation algorithm has a mean dice similarity coefficient of 85.75±1.75%. We found that 28 of 68 extracted imaging features were correlated well with SUV_max_ (*p*<0.05), and some of the textural features (such as entropy and maximum probability) were superior in predicting morphological changes of radiotracer uptake regions longitudinally, compared to single intensity feature such as SUV_max_. We also found that integrating textural features with SUV measurements significantly improves the prediction accuracy of morphological changes (Spearman correlation coefficient = 0.8715, *p*<2e-16).

## Introduction

Positron Emission Tomography (PET) is a non-invasive functional imaging method that captures the distribution of biologically targeted radiotracers at the molecular level, with high sensitivity [1]. Standardized uptake value (SUV) is often used in clinical PET imaging as a semi-quantitative, functional measurement of radiotracer activity, normalized for dose and body weight (or lean body mass or body surface area). Recent investigations have aimed to improve the characterization of radiotracer uptake patterns in order to analyze lesions [2]–[5]. These efforts to characterize patterns of uptake are based on the limitation of SUV measurements such as inconsistent cut-off values for discriminating benign and malignant activity, partial volume effects, body composition, and habitus. Note that SUVs are linearly related to image intensities through patient and scanner specific parameters as well as kinetics of the radiotracer. Although parametrically related, different formulations of SUVs (i.e., SUV_max_, SUV_mean_, etc) are used to overcome the current limitations of SUV measurements [3], and comprehensive analyses of local to global textural and shape characterization of uptake regions remain unaddressed. Extracting characteristic texture/shape features from uptake regions require robust, accurate, and reliable medical image segmentations; however, primarily due to overlap or close juxtaposition of abnormal signals, with surrounding normal structures, background radiotracer activity, image reconstruction-based artifacts, partial volume effects, low resolution, etc., the PET image segmentation can be a challenging problem. Many studies using segmentation of PET images are performed using manual approaches—fixed-, adaptive-, or iterative-thresholding—and region based methods such as fuzzy c-means (FCM), region growing, or watershed segmentation methods [6]–[10]. However, all these methods have limitations in clinical practice because of the following restrictions: (i) desired physical accuracy is usually far beyond the outputs of the methods, particularly for small lesions and uptakes with non-spherical shapes, and (ii) robustness and reproducibility of delineations are two unsolved problems in segmentation of uptake regions from PET images because an algorithm working in different signal-to-background ratio conditions—with similar performance and outputting the same/similar results consistently—is missing.

Our aims in this study are to explore imaging features that may potentially drive morphological characterization of radiotracer uptake and reliably predict morphological changes of abnormal regions. Our investigation produced a robust, accurate, and efficient image segmentation method, which enables a comprehensive texture analysis possible. The relationship of both textural and shape features to intensity based (i.e., SUV) features were also analyzed using multivariate and Bayesian statistics. In this paper, we present the theoretical analysis of textural characterization and image segmentation methods, in addition to experimentally demonstrating that the proposed texture based features—extracted from accurately delineated radiotracer uptake regions—can potentially be used as semi-quantitative tools in analyzing longitudinal morphological change analysis. The combination of SUV_max_ and the proposed textural features are hypothesized to predict morphological changes of abnormal regions more efficiently. The proposed methods were used to detect and identify lung abnormalities, pertaining to patients who had PET-CT scans and histopathology biopsy. Longitudinal analyses of these patients were used to evaluate the ***generalizability*** and ***consistency*** of the proposed method. Although changes in uptake or SUVs can be used as a quantitative index for treatment responses, in this study we confine ourselves into only morphological changes and prediction of these changes in image space with the aim of developing a quantitative and reliable computational platform.

## Methods

### Patients and PET-CT Imaging

With IRB approval, we collected 60 ^18^F-FDG-PET imaging scans from 30 patients. The study population consisted of 12 males and 18 females, with a mean age of 48±12.6 for female (range: 35–75, median: 45 years), 44±14.5 for male (range: 27–64, median: 47 years), respectively. All the patients presented with either primary non-metastatic, metastatic disease, or a systemic viral infection at the time of the first PET scan. The study group consisted of non-consecutive patients diagnosed with primary lung cancer (NSCLC and SCLC), diffuse large B-cell lymphoma (DLBCL), metastatic papillary renal cell carcinoma, cerebellar hemongioblastoma, neurofibroma, lymphomatoid granulomatosis, lung neoplasm, neuroendocrine tumor, soft tissue thoracic mass, nonnecrotizing granulomatous inflammation, renal cell carcinoma with papillary and cystic features, or metastatic alveolar soft part sarcoma. All 30 patients underwent an ^18^F-FDG-PET/CT protocol, where patients were instructed to fast for a minimum of 6-hours before scanning. The serum glucose level was measured to ensure that the value was less than 118 mg/dL (6.5 mmol/l). At the end of the 6 hour period, 321.9–395.9 MBq (8.7–10.7 mCi, median 10.2 mCi) of ^18^F-FDG was administered intravenously to the patients, followed by a 45–60 minute uptake period, before image acquisition (mean uptake period = 54.5 mins, minimum uptake period = 45 mins, maximum uptake period = 60 mins). For the analysis of longitudinal studies, the deviation of ^18^F-FDG uptake periods between the baseline and follow-up scans must be within +/− 10 minutes [11], and our study had a mean deviation of less than one minute. The ^18^F-FDG uptake period deviation between the baseline and follow-up scans was as follows: 22 patients less than 1 min, 7 patients approximately 2 mins, and only one patient had a difference of 4 mins; hence, no significant differences were observed in uptake times between baseline and follow-up scans. Moreover, mean variation of administrated ^18^F-FDG (over all patients) between baseline and follow-up scans was measured as 1.05 mCi. PET images were acquired with 2–3 minutes of emission scan per bed for 5–6 bed positions with 3D acquisition mode. Corresponding non-diagnostic low dose CT was obtained for attenuation correction and anatomic localization. PET-CT Images were collected in two different time points (baseline and follow-up; mean time interval between scans was 267 days, median: 206 days, ranging from 64 to 719 days with multiple scans). The images were 150×150 pixels resolution, corresponding to 4 mm×4 mm pixel size and 4 mm slice spacing. Each patient's baseline and follow-up scan was carefully analyzed, and during the computational and SUV based analysis, up to five lesions were taken into account and tracked longitudinally (Table 1). Since not all patients were having multiple lesions, in order to avoid any bias towards small/big size or regular/irregular shaped lesions, we tracked as many lesions as possible from patients for longitudinal quantification. Follow-up scans of patients were obtained immediately after five chemotherapy cycles to be consistent in the evaluations, and we used the response evaluation criteria in solid tumors (RECIST) since it suggested the use of five lesions per organ (up to maximum 10 lesions) for analysis. Note also that, patients having secondary severe symptoms and complications such as kidney failure during these five cycles were not included in the selection procedure and hence in the study.

**Table 1:** Patient demographics with gender information; SUV_max_ values corresponding to lesion numbers (denoted by L#) both for baseline and follow-up scans are enlisted.

Patient Info.		SUV_max_ (baseline)					SUV_max_ (follow-up)				
ID/Disease	Gender	L1	L2	L3	L4	L5	L1	L2	L3	L4	L5
**1/lung neoplasm**	M	2.2	2.5				2.6	2.9			
**2/Renal cell carcinoma with papillary and cystic features**	M	2.5	3.3				2.1	3.3			
**3/SCLC**	M	5.4	2.1	2.1			8.9	4.1	2.3		
**4/Soft tissue thoracic mass**	F	8.2	7.9	3.5	1.5	1.2	3.8	3.7	1.6	1.0	1.8
**5/SCLC**	F	1.0	5.4				4.9	2.0			
**6/cerebellar hemongioblastoma**	M	3.5	3.5	9.0	2.4		4.5	3.2	14.1	3.5	
**7/DLBCL**	F	5.2	3.0	4.2	6.2	6.7	7.0	3.0	4.2	6.2	4.5
**8/nonnecrotizing granulomatous inflammation**	F	4.2	14.2	5.0			4.3	10.5	1.0		
**9/Soft tissue thoracic mass**	F	8.3					5.0				
**10/NSCLC and squamous cell carcinoma**	M	2.5	8.1	1.1			2.6	8.3	1.5		
**11/lymphomatoid granulomatosis**	F	4.3	5.5	10			1.6	7.7	1.7		
**12/lymphomatoid granulomatosis**	M	1.8	2.5	2.7			1.0	1.0	1.1		
**13/metastatic alveolar soft part sarcoma**	M	4.3	1.3				4.3	1.1			
**14/neurofibroma**	M	8.6					1.9				
**15/NSCLC**	F	7.5	7.5	5.8	4.0	2.9	9.5	9.1	5.7	4.4	1.0
**16/NSCLC**	M	5.5	11.2	2.0	6.0		1.1	7.5	3.3	5.6	
**17/lymphomatoid granulomatosis**	F	14.1	4.7				10.3	2.5			
**18/NSCLC**	F	11.2	7.5	7.0	1.7		10.8	5.6	1.9	4.9	
**19/Soft tissue thoracic mass**	F	3.4					1.6				
**20/SCLC**	F	7.5	9.3	9.5	10.4		5.0	8.7	8.9	11.7	
**21/NSCLC**	M	5.1	5.0	4.8	11.5	12.0	1.2	1.1	1.0	4.1	4.5
**22/SCLC**	F	1.1	1.9	2.4	2.1	3.1	2.5	2.5	2.5	3.5	3.0
**23/NSCLC**	F	7.5	5.5	3.5			5.1	2.2	1.0		
**24/metastatic papillary renal cell carcinoma**	F	1.7	7.4	5.2			3.6	9.1	6.6		
**25/DLBCL**	M	3.5	8.7				3.3	7.5			
**26/neuroendocrine tumor**	F	3.7	2.2	3.5	1.7		2.7	2.3	1.0	1.1	
**27/SCLC**	F	4.6	4.0	4.1	3.7	1.2	5.0	4.1	4.1	3.5	1.0
**28/SCLC**	F	2.5	14.0	6.7	6.5	6.9	7.0	8.0	8.3	6.7	6.7
**29/NSCLC**	M	7.5	2.2	1.0			7.4	4.3	4.1		
**30/neuroendocrine tumor**	F	9.0	6.6	5.7			8.5	7.0	1.0		

Number of lesions is subject to change from patient to patient.

### Analysis of Uptake Regions Using Textural and Shape Features

**Figure 1:**
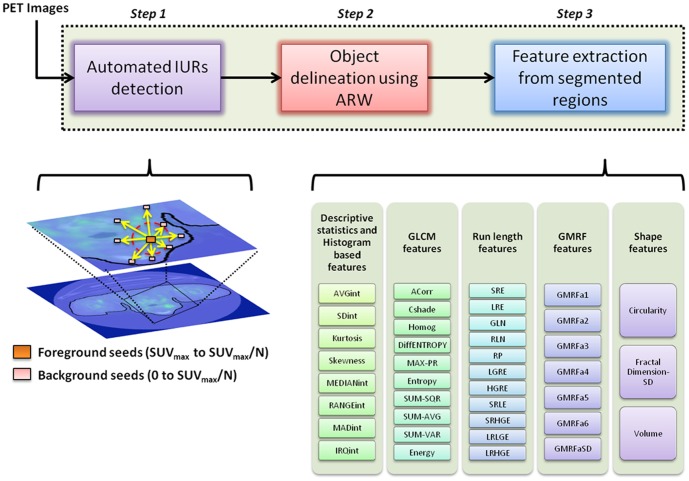
Average intensity (AVGint), standard deviation of intensities (SDint), median value of intensities (MEDIANint), maximum absolute deviation of intensities (MADint), interquartile of intensity histogram (IRQint), Autocorrelation (ACorr), contrast shade (Cshade), homogeneity (Homog), difference of entropy (DiffEntropy), maximum probability (MAX-PR), summation of square of intensity values (SUM-SQR), summation of average of intensity values (SUM-AVG), summation of variation of intensity values (SUM-VAR), short run emphasis (SRE), long run emphasis (LRE), gray level non-uniformity (GLN), run length non-uniformity (RLN), run percentage (RP), low gray level run emphasis (LGRE), high gray level run emphasis (HGRE), short run low gray level emphasis (SRLE), short run high gray level emphasis (SRHGE), long run low gray level emphasis (LRLGE), long run high gray level emphasis (LRHGE).

Texture analysis provides quantitative information describing properties in images such as coarseness and smoothness. The search for useful textural features and discriminative statistics in image processing field has significantly progressed throughout the last three decades [12]. Co-occurrence matrices [13], run-length statistics [14], local shapes [15], and cliques in Markov random fields [16], as well as many extensions of these landmark features are well-established in various disciplines. Parallel to these developments, in recent publications, textural and shape features of uptake regions were used to characterize esophageal cancer in [3], human sarcomas in [15], cervix, head and neck cancers in [4]. In particular, local tissue characteristics provided by PET and modeled by textural heterogeneity—by computer algorithms—were explored to understand the biological function of different tissues. However, in practice, the aforementioned computational methods used for analyzing functional uptake in PET images do not provide a general way for reliable inference, due to the highly possible segmentation errors and difficulties in characterization of global and local features. Note also that inaccurate delineation of uptake regions may cause to considerable changes in extracted features. Last but not least, local variations of feature values were usually ignored or not taken into account in such studies [5], [13], [14], [17]–[19]. However, we postulate that local variations of feature values might be more effective than the features themselves in terms of correlation levels. In this study, we addressed all of these problems in two steps: (i) by proposing a robust, accurate, and fast segmentation method, as described in next subsection, and (ii) by broadly and deeply analyzing different textural and shape features, as well as their local deviations, from accurately delineated regions. Figure 1 shows feature types and associated features, extracted from delineated uptake regions from PET images. Brief descriptions of the features are explained in the following subsections.

### Automated Random Walk (ARW) Image Segmentation

When images are low resolution and include noise, graph based segmentation algorithms were shown to be more useful than boundary and thresholding based segmentation methods [20]–[23]. PET images, as a nature of the reconstruction process, are low resolution images with high contrast and include noise; therefore, graph based segmentation algorithms are more suited for radiotracer uptake segmentation. We used an adaptive graph theoretic segmentation algorithm—automated random walk (ARW) image segmentation—in order to produce automated, efficient, and reproducible object delineation results from PET images. ARW works as follows: first, object and background are roughly identified by using an automated interesting uptake region (IUR) algorithm, and then some voxels are labeled as either object or background region, accordingly. Second, the delineation algorithm is initiated to efficiently and quickly determine the label of the remaining unlabeled voxels. The proposed ARW determines the highest probabilities for assigning labels to voxels, by measuring the “betweenness/togetherness,” by initiating random walkers from a labeled voxel, and by reaching to the unlabeled voxel first by a random walker. The proposed method is different from the conventional random walk algorithm [24] in the following ways: (i) the proposed method is fully automated since it detects interesting uptake regions (IUR) automatically, and (ii) the proposed algorithm is performed based on the SUVs of voxels, and *prior probability distributions* of voxel SUVs were calculated using a robust kernel density estimation method [25] instead of using simple Gaussian assumptions. For (i), we automatically localized the seeds for object and background separation, based on the high contrast difference of PET images. We accomplished this identification step by defining an encoder function *c* (see equation 1.1), which is a threshold interval for PET images:
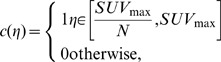
(1.1)where 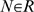 and 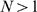. Regions identified by the encoding function were considered as IURs. Once IURs were identified for each IUR, the voxels with the SUV_max_ of that particular IUR were marked as foreground seeds (i.e., SUV^IUR^_max_). Then, we explored its neighborhood through 8-connectivity graph labeling algorithm [26] to find voxels with values less than and equal to the SUV_max_/*N*, where *N* is pre-defined value greater than 1. Those voxels were marked as background seeds. Once foreground and background seeds were localized (i.e., automatic detection step), random walk image segmentation was initiated by these inputs. In all experiments, *N* was set to 2.5 as equal to the conventional clinical usage (i.e., 40% of SUV_max_ is usually selected as thresholding value) [2]. For (ii), instead of using the pure intensity values of voxels, we adapted SUVs of voxels in ARW algorithm. In addition, during the computation of prior probability distributions of labeled (i.e., localized seeds) voxels, we used an adaptive kernel estimation method [25] to accurately compute the priors even though the number of labeled voxels were small. In the proposed detection approach, it is important to emphasize that the foreground seeds are localized based on the highest intensity values (i.e., SUV_max_), whereas background seeds are localized with respect to the foreground seeds through a search algorithm. Since random walk segmentation only needs a few cues for foreground and background, and it is quite robust to a leaking problem—commonly seen in graph cut algorithms—a “rough” identification of the parameter *N* is sufficient to finalize the seeding process. Note also that segmentation as a whole can be considered as consisting of two related tasks: recognition and delineation. Recognition is the process of determining roughly “where” the objects are, and it distinguishes them from other object-like entities in the image, while delineation is the final step for defining the spatial extent of the object region/boundary in the image. This recognition task coincides well in our detection algorithm, which roughly identifies IURs and feeds this information to random walk delineation to make it fully automated. Additional information and experimental validations on automatic detection of IURs can be found in **Appendix S1**.

#### Random Walks for Image Segmentation

Lets represent an image as a weighted undirected graph (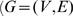, 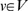 and 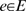), with its nodes/vertices (v_i_) as voxels and edges (e_j_), defined as voxel adjacency with cost values assigned to edges (w_ij_). We used the un-normalized Gaussian weighting function to define edge weights as 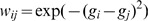, where g_i_ represents the SUV of voxel *i*. By the convention of detected IURs, some of the vertices of the graph were known (denoted by V_M_), and some were not known (denoted by V_U_), such that 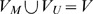 and 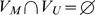. The segmentation problem was reduced to finding labels of unlabeled vertices (nodes). A combinatorial formulation of this situation could be written as a Dirichlet integral as:
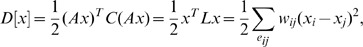
(1.2)where C was the diagonal matrix with the weights of each edge along the diagonal, and A and L( = A^T^CA) were incidence and Laplacian matrices indicating combinatorial gradients, and defined as
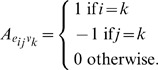
(1.3)


**Figure 2:**
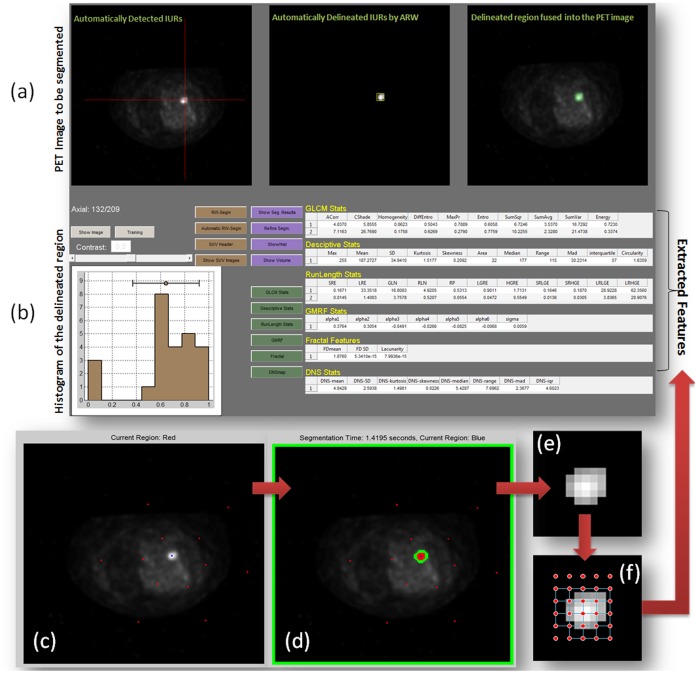
An example view from our proposed framework/software is shown. (a) Automatically detected uptake regions (first), its segmented version (second), and fusion of segmented region into original image (third) are shown. After detection, the details of object (blue) and background (red) seeding and conducted segmentation are shown in (c) and (d), respectively. Segmented region (e) is divided into local windows and for each local window pre-defined textural and shape features are extracted (f). Tools to control extraction of textural features, segmentation, SUV analysis, and the immediate results are shown in (b).

**Figure 3:**
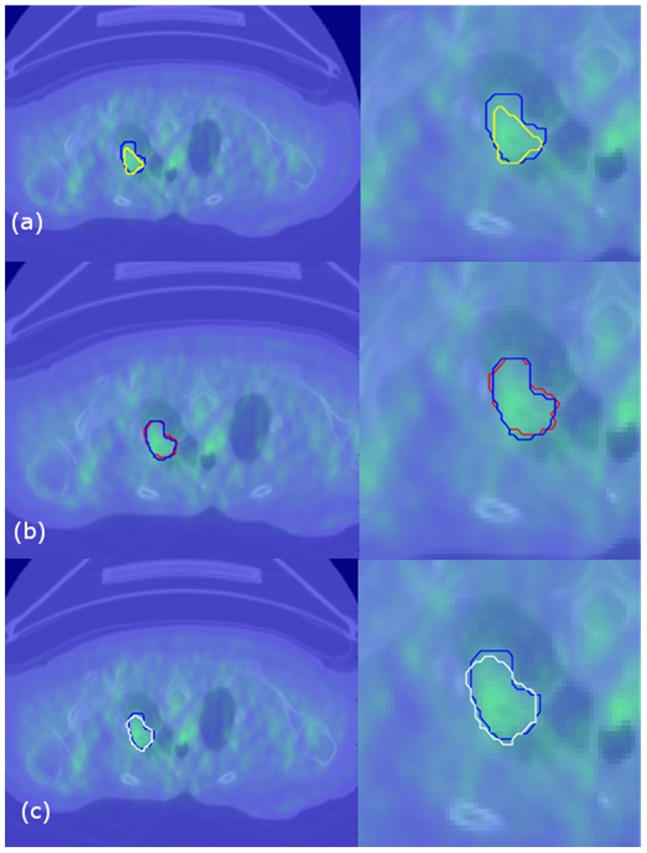
First row: an example inter-observer variation was demonstrated in fused PET-CT image (user 1: yellow, user 2: blue drawings). Second row: an example intra-observer variation was demonstrated (user 1 time 1: blue, user 1 time 2: red drawings). Third row: Users drawing (blue) and automatically found (white) boundaries of uptake regions were demonstrated.

**Figure 4:**
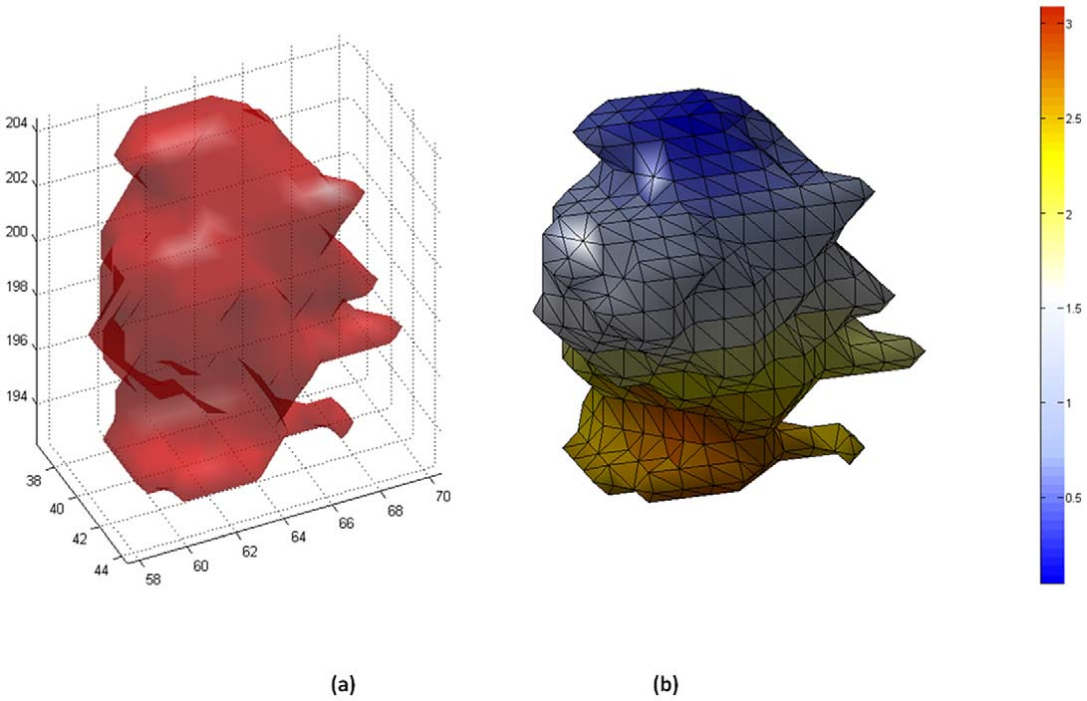
An example surface pair obtained from segmented uptake regions (i.e., non-specific mass from lung regions of a particular patient) is shown. We parameterize the surface (a) of lesion using Euler angles of boundary points, and we colorized the surface points with respect to those angles in radians (b). This shape information (i.e., circularity) was used in longitudinal assessment of uptake changes.

The solution of the combinatorial Dirichlet problem may be determined by finding the critical points of the systems. Differentiating D[*x*] with respect to *x* and solving the system of linear equations with |V_U_| unknowns yielded a set of labels for unlabeled vertices. Figure 2 (a and b) revealed a general view of our proposed software, where any selected slice of PET scan (Figure 2a) automatic detection of IURs was completed prior to delineation, and delineated regions (Figure 2b) were fused into original gray scale image (Figure 2c). Details of this process are exemplified in Figure 2 (c,d,e). A few voxels belonging to radiotracer uptake regions and background (Figure 2c) were detected automatically in the first step, and resultant ARW delineations are shown in Figure 2d. Texture and shape information were extracted from those automatically delineated regions (see extraction of features part in Figure 2b). Some delineation examples from ARW methods (Figure 3b, blue boundary) and inter-and intra-observer variations (Figure 3a and Figure 3c, red boundary), respectively, were overlaid in a two-dimensional form for comparison. In addition, Figure 4 a and b show surface information of delineated object both in object rendering and parametric surface modes.

### Feature Extraction

#### Descriptive Statistics and Histogram Based Features

Descriptive statistics measure the likelihood of observing a gray value at a chosen location in the image. The average, maximum, minimum, standard deviation (SD), median and range intensity values are some of the examples for descriptive statistics. All of these statistics can be derived from the histogram of voxel intensities in the images. Further characterization of the data variability can also be handled by incorporating higher-order statistics into the histogram analysis. For example, some of the histogram based features such as skewness, kurtosis, median absolute deviation (MAD), and interquartile range (IRQ) provide a natural bridge between images and a probabilistic description; however, estimation of a density profile from experimental data points is a challenging issue, especially because the number of data points is limited. Considering the studies that used histogram-based features for textural characterization of radiotracer uptake regions, accurate estimation of histogram features is often not possible. Therefore, we derived a histogram based on global features of textural regions, through kernel density estimation with diffusion approach [25]. This approach is an accurate and reliable non-parametric method, and it is able to deal with a small number of data points. Another important contribution that we have made was to capture local variations of global features. Since it is a well-known fact that a region in an image has a constant texture—if a set of local statistics or other local properties of the picture function are constant—slowly varying, or approximately periodic [19], it was thus of interest to provide global statistics in a local sense in order to discriminate and characterize textures of region of interest. To achieve this, we extracted descriptive statistics and histogram based features from local patches (see Figure 2e), which we obtained after dividing automatically delineated radiotracer uptake regions into certain size non-overlapping blocks (i.e., 3×3, 5×5, 7×7, 9×9, and 11×11 pixels size blocks were used, and the best block size was found to be 7×7 pixels and non-overlapping). We extracted all features from 2D sections of segmented 3D objects slice-by-slice and concatenated them (i.e., pseudo-3D) in a feature extraction order to avoid an additional slice sampling load and possible partial volume effects. The best window size was selected based on the highest value of the summation of mutual information (i.e., maximum mutual information: MMI) values over all local windows. Thus, we extracted global features in a local sense, and we computed the variations of these features over all the local regions (i.e., we had additional feature sets derived by computing standard deviation of computed global features such as SD of average intensities, SD of MAD, SD of IRQ, SD of kurtosis, etc.).

#### Gray Level Co-occurrence Matrix (GLCM) Based Features

Descriptive statistics and histogram features depend on individual voxel values and not on the interaction or co-occurrence of neighboring voxel values; therefore, they suffer from the inability to encode spatial image variation. Since GLCM based features, in this sense, are second order statistics—estimating the spatial distribution of gray levels—GLCM based feature extraction methods have become one of the most well-known and widely used textural feature extraction methods for various different aims [13]. Some of the GLCM features used in our system included entropy, correlation, contrast, etc. The full list of features are listed in Figure 1. Entropy feature, for example, measures the amount of uncertainty (disorder) in the image. On the other hand, the *maximum probability* feature (MAX.PR) measures summation of the likelihood of voxels having the most common value for a given region. GLCM features help extract complex image properties by considering spatial variations of voxels pertaining to particular regions of interest. However, in most of the literature about radiotracer uptake characterization, not only the local deviations of these features were ignored, but also the optimal window size for extracting local and global features was not investigated. To tackle this problem, we divided the uptake regions into local regions, as explained in the previous subsection, and then we found the best window size for local and global analysis, by conducting correlation analysis of local regions inside the uptake regions (i.e., for different pre-defined window sizes, highest correlation value obtained among local regions was used to select the best window size). We then incorporated the local standard deviations of the extracted features into our proposed system for further characterization of the uptake regions. In the results section, we demonstrated that some of the textural features have lower correlations with SUV_max_ than their variations.

#### Run-Length Features

Run-length method is an effective texture analysis approach which examines the coarseness of a texture in a specific direction (i.e., number of runs with voxels of a particular gray level) [18]. Various texture features are derived from this information such as short run emphasis (SRE) or long run emphasis (LRE), etc. Basically, run length features are determined for the segmented image regions by taking into consideration the heterogeneity of these regions. The statistical properties of the run of a particular gray level in an image are significantly influenced by the size of the segmented regions; therefore, unlike the other studies reported in the literature [2], [3], [12], [18], we adaptively selected the window size for analysis of the runs by examining the highest autocorrelation between different size of the local windows and probability distribution of each gray level's run-length feature. Figure 1 shows the complete list of run-length features used in our broad analysis. Note that it has been shown here and in the literature [18] that run length features possess as much discriminatory information as conventional texture features such as GLCM features. Please see [4], [18] for technical details and further explanations on run-length features.

#### Gaussian Markov Random Field (GMRF) Features

Most medical images are Markov Random Field (MRF) images, that is, the statistics of a voxel in the medical image that are related to the statistics of voxels in its neighborhood [27], [28]. A challenging problem in extracting suitable features from images is to extract robust features that are invariant to rotation and scaling. For instance, although multiple tumors with the same pathological findings may have different size and location within the image, extracted textural features are desired to have similar values that are independent of their size and location if characterization by texture is aimed. MRF, in this case, may offer a solution to this problem by providing a powerful tool to model the probability of spatial interactions in an image. By incorporating Gaussianity assumption to the MRF framework, we were able to extract rotation and scale invariant textural features from segmented uptake regions [17]. GMRF model was defined by the following equation [17], [27]:
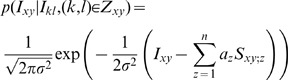
(1.4)where the equation denotes the probability of a voxel (x,y) having a specific gray value I_xy_ given the values of its neighbors, n (n = 6 in this particular study) is the total number of pixels in the neighborhood Z_xy_ and S_xy;z_denotes the summation of two symmetric pixels. We estimated the GMRF parameters (i.e., 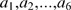 and 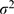) by using a *least square error estimation method*, similar to the study in [17].

#### Shape Features

The local relationship between fuzzy/solid objects and the intensity distributions—pertaining to those objects—is obtained through shape (geometric) features. There have been some techniques explored in [5], [15] for evaluation of ^18^F-FDG-PET utilization characteristics in human sarcomas such that a measure of heterogeneity incorporating ***tumor shape information*** was shown to be superior, compared to a measure of heterogeneity alone. Similarly, we encoded the 2D/3D boundaries of segmented regions and computed the “circularity/sphericity” of those regions, as well as fractal geometry and volume information of those regions. These features were extracted from the 3D segmented radiotracer uptake regions. Extracted features were used to explore the correlation between functional information and the anatomical boundary of functional uptake. While volume (V) was computed by multiplying the voxel size with the number of voxels occupied in the uptake region, circularity (or sphericity in other words) is calculated as 
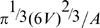
, where A denotes the surface area of the segmented region (i.e., voxels interior to the segmented objects are not counted in surface area computation). Sphericity measures disparity between the shape of an object and a perfect sphere (i.e., roundness). In addition, we also extracted fractal geometry of 3D segmented regions, where the fractal was defined as an object with the self-similarity property, i.e., it appears the same at different magnifications. Fractal measures are frequently used to understand underlying phenomena in different biomedical applications, including the cancer diagnosis. It provides information on the regularity and complexity of an object by quantifying its self-similarity level. We measured the fractal properties of the segmented objects by the box-counting method, as described previously in [17], [29].

## Results

### Evaluation of Segmentations

The dice similarity coefficient (DSC) [30] and Hausdorff distance (HD) [31] were used to evaluate segmentation accuracy, with respect to ground truth (i.e., surragate truth) provided by expert's manual delineations. Note that we use the term ground truth and surrogate truth interchangeably. Also, since our analysis includes only PET images, lesion volume should be regarded as functional volume only (functional volume is not necessarily equivalent to the tumor volume). Indeed, true tumor volume can only be validated with histopathology. While DSC is a measurement of spatial overlap (in percentage) between segmented object (lesion) and surragate truth (manually delineated lesion by experts), HD is a shape dissimilarity metric measuring the most mismatched boundary points between the segmented object and ground truth. High DSC and low HD values indicate goodness of the image segmentation method. Furthermore, we also analyzed inter-and intra-observer variations by DSC overlap ratios, since simple Pearson correlations can be misleading [32] (i.e., segmented volumes may have the same values although volumes may not overlap or overlap very little). Two expert radiologists delineated radiotracer uptake regions in three different time points (one week between each drawings, and blinded to each other's drawings). Each expert's drawings—in different time points—were used to compute intra-observer agreement ratios. Table 2 summarizes the evaluation of segmentation results for the proposed method compared to mean and individual delineation definitions of experts, as well as inter- and intra-observer agreements. Evaluation metrics (DSC and HD) are formulated and described in detail in the **Appendix S1**.

**Table 2:** Evaluation of the proposed segmentation methods (via DSC and HD) and observer agreement ratios are given.

Evaluation Criteria	Values
**Computer & Observer 1 DSC (%)**	84.50±3.21
**Computer & Observer 2 DSC (%)**	87.01±0.32
**Mean DSC (%)**	85.75±1.76
**Computer & Observer 1 HD (mm)**	4.90±0.47
**Computer & Observer 2 HD (mm)**	5.45±0.52
**Mean HD (mm)**	5.18±0.50
**Inter-observer agreement DSC (%)**	77.73±6.49
**Inter-observer HD(mm)**	10.50±1.30
**Intra-observer agreement DSC (%)**	89.86±4.23
**Intra-observer HD(mm)**	3.90±1.50

### Exploring Connections among Extracted Features

**Figure 5:**
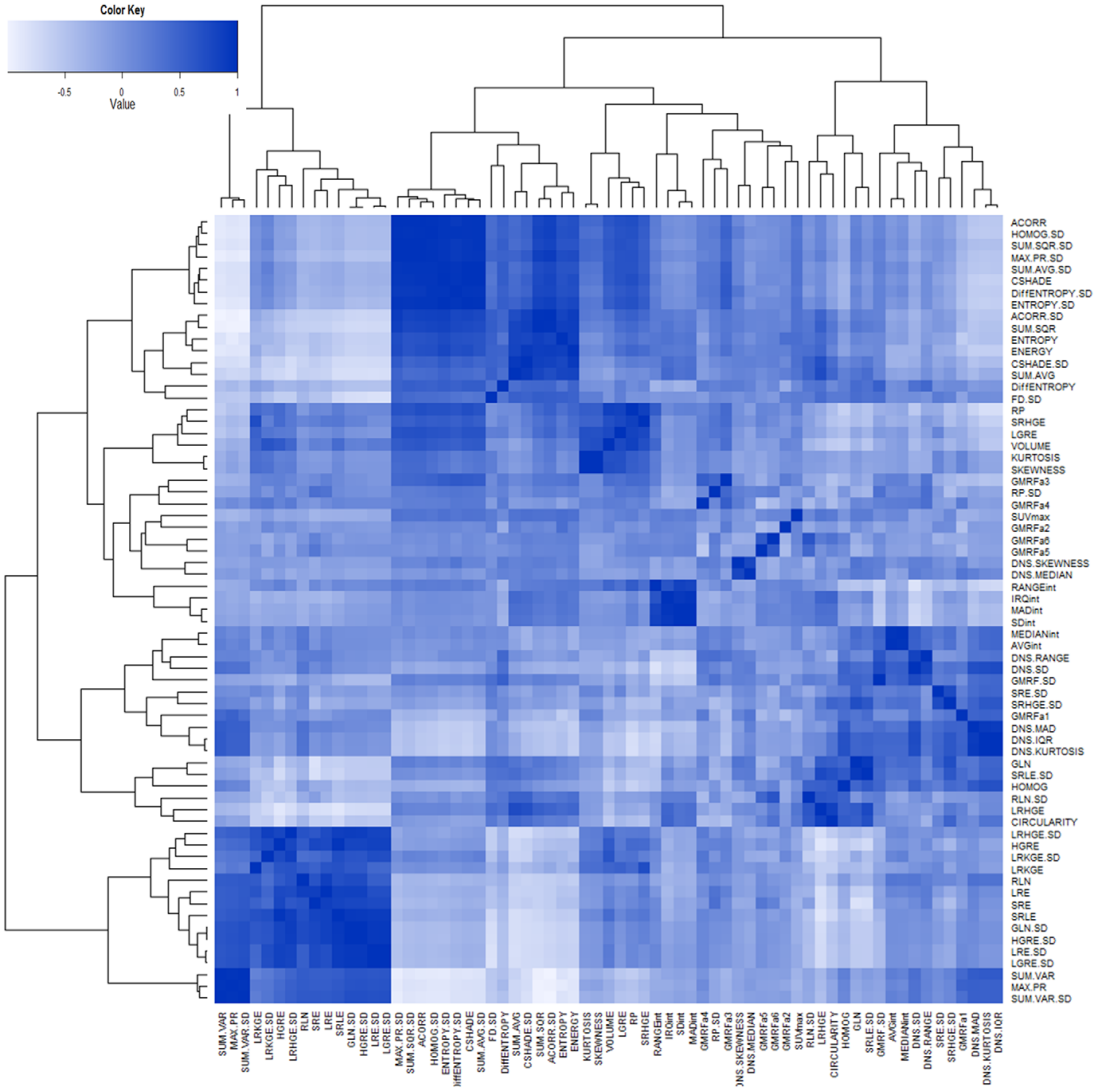
Hierarchical clustering is used based on the correlation of all data at hand. A correlation matrix together with clustering (i.e., Pearson uncentered) of the feature points is presented from R = −1 negative correlation (white) to R = 1 positive correlation (blue).

**Figure 6:**
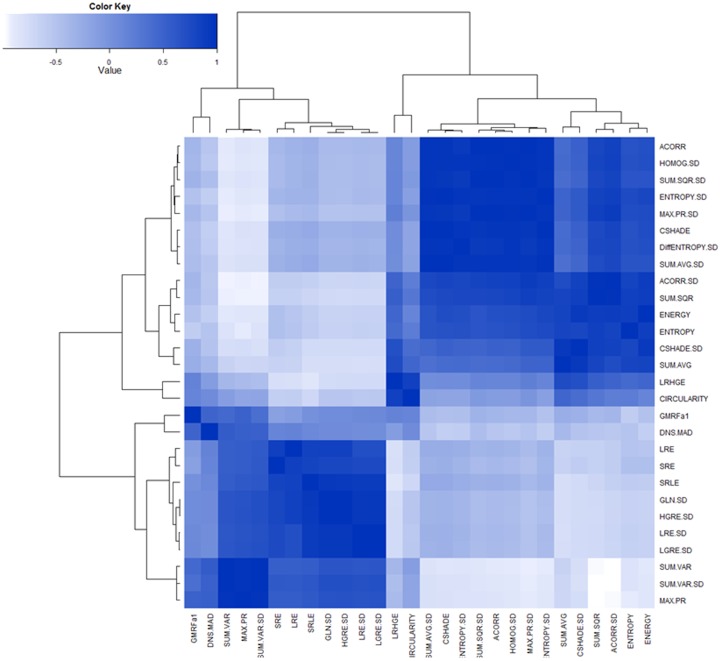
Correlation matrix is obtained using only the features, which have strong correlation with SUV_max_ features. The resultant correlation values are used in hierarchical clustering algorithm to show the detail relationships of the feature sets. Again correlation values are running from R = −1 negative correlation (white) to R = 1 positive correlation (blue).

We integrated all extracted texture, shape, and SUV_max_ features into an unsupervised hierarchical clustering algorithm [33]. Our aim was to explore similarities and dissimilarities of features and to clarify hidden connections among features that can be integrated together in order to more accurately predict radiotracer uptake region morphological properties (without conducting any claim about clinical utility of these features) such as change in volume and shape (i.e., morphological characterization). The presence of clusters in a data set is frequently due to the existence of certain relationships between measured variables. Moreover, true group (class) membership is unknown to these variables. We conducted unsupervised clustering of the measured variables in order to explore true (or surrogate true) memberships. Euclidean distance dissimilarity measure with complete leakage method [34] were used to find highly correlated features and to contain them in similar clusters. Figure 5 demonstrates correlation analysis of all feature sets through a correlation matrix (whose column and rows shows the features), and dendogram graphics (i.e., hierarchical tree structures) for each feature were integrated into columns and rows of the correlation matrix. Similarly, we extracted the hierarchical tree structures only for features that have statistically significant correlation values with SUV_max_ values. The resulting clustering scheme is illustrated in Figure 6. We repeated the same step for each type of feature separately (run-length, GLCM, etc., as shown in Figure 5 and 6) to clarify if the features were coming from significantly different class (membership) or not (from R = −1 (white) to R = 1 (dark blue), Figure 6). In particular, since SUV_max_ is the current standard in quantification of uptake regions, we computed the Pearson correlations of all features with SUV_max,_ and we reported only significantly correlating features in Table 3; however, one may introduce different quantification features to repeat this task. Among all features having significant correlations with SUV_max_, it is interesting that none of the features share the same cluster that SUV_max_ occupies, that is, those features are found to be informative in a semi-quantitative sense like SUV_max_ itself. Another potentially important finding, observed in Table 3 and Figure 5, was that the standard deviation (SD) of some features—most of the GLCM features and some of the run-length features (i.e., LGRE.SD, GLN.SD, HGRE.SD, etc.)—were outperforming the features in correlation measurements. Note that some SD based features are coming from the local approach that we follow in the feature extraction. In Table 3, compared to the local approach, we also demonstrated the performance of global approach for textural analysis where, features were extracted from the segmented regions, without taking into account the local variations of the features within the scene. It is evident with this finding that not only do global heterogeneity of spatial features provide better associations among features, but local heterogeneity (SD) of both spatial and shape features also provide better correlations, as agreed with our initial assumption.

**Table 3:** Correlation ratios, 95% confidence intervals, and p-values of significantly correlating textural features with SUV_max_ are summarized.

*Features*	*Correlation Ratio (R)– Local Approach*	*95% Confidence Interval (CI) [Min and Max]*		*p*-value <0.05	*Correlation Ratio (R)-Global Approach*
**SRE**	−0.342	−0.540	−0.100	0.005	−0.146
**LRE**	−0.293	−0.500	−0.046	0.015	−0.125
**SRLE**	−0.292	−0.499	−0.044	0.016	−0.125
**LRHGE**	0.307	0.067	0.522	0.016	0.131
**LRE.SD**	−0.235	−0.452	0.016	0.049	--
**GLN.SD**	−0.282	−0.491	−0.033	0.019	--
**LGRE.SD**	−0.238	−0.454	0.013	0.048	--
**HGRE.SD**	−0.284	−0.492	−0.035	0.019	--
**CIRCULARITY**	0.254	0.010	0.479	0.049	−0.127
**GMRFa1**	−0.299	−0.505	−0.052	0.013	−0.299
**DNS.MAD**	−0.288	−0.496	−0.040	0.017	−0.288
**ACORR**	0.323	0.084	0.535	0.011	−0.235
**CHSADE**	0.341	0.104	0.550	0.007	−0.247
**MAX.PR**	−0.363	−0.556	−0.124	0.003	0.264
**ENTROPY**	0.388***	0.158	0.588	0.002	−0.283
**SUM.SQR**	0.351	0.115	0.558	0.006	−0.255
**SUM.AVG**	0.324	0.085	0.536	0.011	−0.236
**SUM.VAR**	−0.328	−0.528	−0.084	0.007	−0.240
**ENERGY**	0.358	0.123	0.563	0.005	−0.265
**ACORR.SD**	0.344	0.107	0.552	0.007	--
**CSHADE.SD**	0.364	0.130	0.568	0.004	--
**HOMOG.SD**	0.295	0.053	0.512	0.021	--
**DiffENTROPY.SD**	0.303	0.063	0.519	0.018	--
**MAX.PR.SD**	0.345	0.109	0.553	0.007	--
**ENTROPY.SD**	0.360	0.126	0.565	0.005	--
**SUM.SQR.SD**	0.318	0.079	0.531	0.013	--
**SUM.AVG.SD**	0.334	0.096	0.544	0.009	--
**SUM.VAR.SD**	−0.319	−0.521	−0.074	0.008	--

*** denotes the highest correlation ratio. Correlation ratios with conventional global approach are also shown in the last column as a comparison to local approach.

### Correlation of Textural, Shape, and SUV_max_ Features and Impact on Morphological Change Predictability

**Figure 7:**
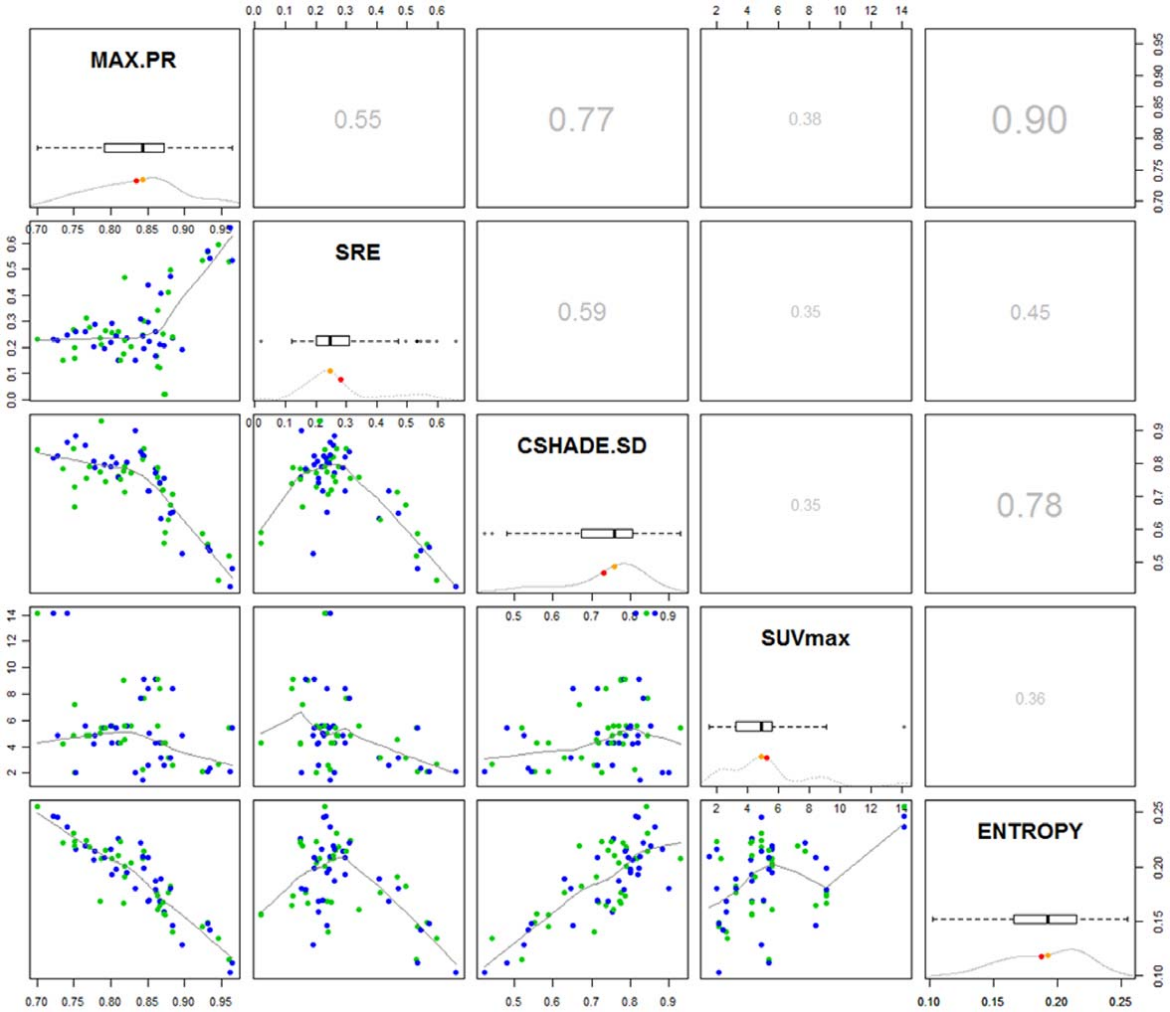
The selected five best informative features, and their histograms, box-plots with mean and median (diagonal), one-to-one regression curves in matrix row-column format (in lower panel), and spearman correlation values (in upper panel) are given.

In longitudinal measurements of uptake regions, we tested the prediction power of each extracted texture feature for estimating morphological changes, including volume and circularity. Since morphological changes such as volume and shape may represent disease severity [5], [15], our proposed technique may also be used in clinical tasks for predicting those morphologic factors using texture features combined with SUV. However, this requires a large spectrum of clinical data as well as ground truth from biopsy samples. In addition, we believe that associations of the image based features should be revealed before testing the proposed methodlogy for clinically more involved tasks. Therefore, we confine ourselves in this section to evaluate image based features and their prediction power analysis to build (near-) optimal associations among image features. We used shape features (i.e., circularity in particular) as our ground truth to test individual image features, without entirely relying on SUV_max_. In addition, we also added the feature “change in volume of radiotracer uptake regions” into our analysis to explore if there was a correlation with suggested informative features. Table 4 reports the results of an analysis in which textural and SUV_max_ features were jointly and individually considered for possible relation to shape and volume changes longitudinally. We concluded from the results from Table 4 that volume change information does not have significant correlation with SUV_max_; however, textural features correlate well with volume change information. Furthermore, combined SUV_max_ and textural features lead to an increase in the correlation ratios, compared to textural features or SUV_max_ alone. Textural features showed superior correlation ratios to SUV_max_ in all cases. Figure 7 shows histograms, pair-wise Spearman correlations and box-plots of selected five best features having the highest predictability values in patient outcome or changes in uptake region characteristics (i.e., SUV_max_, SD of contrast shade (CSHADE.SD), entropy, maximum probability (Max.PR), and SRE). For multiple variable selection and to use them in morphological change prediction, a simple logit transformation [32] was used so that parameters of the logit transformation were obtained through maximum likelihood estimation method. In order to validate both parameters of logit regression and prediction ability of the combined model, we used a leave one out cross validation (LOOCV) sampling technique. Circularity and volume, on the other hand, were combined through a simple multiplication operation, where lesions having the same *volume/circularity* information were differed from each other with *circularity/volume* information, respectively. In addition, we found that there is no significant volume and circularity differences between ground truth and segmentated lesions as indicated by DSC rates in Table 2. As earlier mentioned, volume correlation of ground truth and segmented sets are only meaningful when they are presented with corresponding DSC rates. Since DSC rates are given in Table 2, then we conducted a *t*-test and Pearson correlation test to find the correlation between volume and circularity measurements of ground truth and computer based calculation. A high Pearson correlation value of R = 0.971, p<0.001 and R = 0.955, p<0.001, for volume and circularity measurements was obtained. Finally, we also determined that the likelihood of these selected features follow normal distribution by using the Shapiro-Wilk test [35]. As a result of this test, entropy and Max.PR features were found to follow a normal distribution, and the rest did not, as summarized in Table 5. To show that Max.PR and entropy follows normal distributions but have significantly different variation, we conducted an F-test [36] between Max.PR and entropy features (F = 0.3042, 95% Confidence interval = [0.1855 0.4987], *p* = 3.98e-6). Note that conclusions about the utility of features were arrived after LOOCV was conducted for all data as usual in supervised machine learning techniques. Once the conclusions were derived with the help of proposed method, for any unseen baseline features, follow-up morphological changes can be predicted.

**Table 4:** Longitudinal assessment of morphological changes of uptake regions through SUV_max_, texture, and combined SUV_max_ and texture features are given with corresponding spearman coefficients and p-values.

CIRCULARITY	Feature	Spearman Coefficients	*p* -value
	**SUV_max_**	0.2713	0.031
	**Texture**	−0.8395	<2.0e-16
	**SUV_max_ & Texture**	−0.8420	<2.0e-16
**VOLUME**	**SUV_max_**	−0.0587	0.70 (ns)
	**Texture**	0.8652	<2.0e-16
	**SUV_max_ & Texture**	0.8715	<2.0e-16
**VOLUME & CIRCULARITY**	**SUV_max_**	−0.0533	0.68 (ns)
	**Texture**	0.8650	<2.0e-16
	**SUV_max_ & Texture**	0.8719	<2.0e-16

Circularity, volume, and combined circularity and volume are used as surrogate truth for morphological change. Prediction abilities of features are summarized with Spearman rank coefficients. ns indicates non-significant correlation, minus sign indicates reverse relationship.

**Table 5:** Shapiro-Wilk normality test for the selected feature is given.

Feature	Test Statistic (W)	p-value
**SUV_max_**	0.86	2.0e-7
**CShade.SD**	0.90	0.0002
**Entropy** *	0.98	0.10
**Max.PR** *	0.98	0.38
**SRE**	0.89	2.0e-5

Entropy and Max.PR follows normality assumption but with different variances (see F-test results in the text).

* indicates accepted null-hypothesis.

## Discussion

^18^F-FDG-PET imaging demonstrates increased metabolism with high contrast, but localization of the radiotracer uptake is limited by low spatial resolution of PET images. Even though the high contrast between tumor and normal tissue on PET images could diminish the variability in tumor regions, observer variability in delineation of tumor is still high with the qualitative use of PET images. Changing the window level could significantly alter the apparent volume and tumor shape; therefore, the qualitative definition of the target volume and well-defined tumor boundaries using PET images is not straightforward and highly dependent on the image interpreter.

Most of the automated abnormal radiotracer uptake delineation methods—in the tissues—have relied exclusively on thresholding an absolute PET intensity value. Both inconsistency in radiotracer uptake among patients and variability of radiotracer uptake in normal and abnormal tissue, within individual patients, influence the performance of these automated methods. Furthermore, these thresholding methods also disregard the “texture” information obtainable on PET images.

One important aspect of our work is that all of the segmentation, feature extraction, and statistical inference methods were based purely on functional PET images. We used this as a hard constraint to maximize the extracted information, and to use this information as a base for the possible incorporation of different information.

Another strong aspect of our feature extraction method is to use adaptive (in discrete sense) window size. A very similar approach for data exploration was recently published by Reshef et al [37]; however, there is also a limitation in this strong adaptive feature extraction method that we proposed here, and similarly in the study of Reshef et al [37]. That is, there is no guaranteed optimal window size for feature selection procedure due to resolution limitation and discreet (and therefore fuzzy) nature of the images—since we are only able to give *near-optimal* solution for window size selection. This is important due to extracting statistically accurate features from uptake regions, and also because of the finding that variability among local windows often carries more valuable information than the extracted feature themselves. This issue is totally new and subject to further investigations under different conditions; for example, different imaging modalities (i.e., MRI and CT) and a variation of SUV measurements.

In this work, we have not discussed the incorporation of anatomical information into the PET functional domain, but rather presented the broad analysis of morphological characterization, both in shape and in spatial space. As an extension of this study, we aim to adapt our feature extraction method to a subject and modality specific framework, where feature extraction methods optimally find the subject specific functional and anatomical information from abnormal regions (i.e., from CT or MRI scans) and corresponding uptake regions (i.e., from PET scans) simultaneously.

## Conclusions

We presented a framework where we automatically segmented radiotracer uptake regions in high accuracy. Our findings show that extracted shape and texture features, as well as SUV measurements from segmented regions, provide broad analysis of morphological characterization of functional information. Our approach produced a unique estimation of morphological features that can be used alone or together with SUV measurements to predict longitudinal changes in volume and shape of uptake regions. We concluded from our experimental results that some of the textural features such as entropy, maximum probability and contrast shading information of local spatial regions, short run emphasis, and variability of these features over different local windows potentially carry the most valuable, and their predictability of morphological change in uptake regions' shape and volume were reported as superior to single intensity based measurements. Integrating the extracted features with SUV measurements may improve our ability to understand the morphological changes of uptake regions over time. We also highlighted how the accurate segmentation expanded our understanding of shape information—extracted from uptake regions—and how well it agreed with the results of landmark studies [5], [15].

## Supporting Information

Appendix S1 (DOCX)
