# Supplementary material for: Predicting Future Morphological Changes of Lesions from Radiotracer Uptake in 18F-FDG-PET Images
Source: PLoS One. 2013 Feb 19;8(2):e57105. doi: 10.1371/journal.pone.0057105 (PMC3576352; doi:10.1371/journal.pone.0057105)
Supplement: Appendix S1 — (DOCX) [file pone.0057105.s001.docx]

# SUPPORTING INFORMATION

Formulation for DSC and HD Evaluation: Given that segmented volume (or set) is denoted by *U_1_* and the ground truth segmentation is shown by *U_2,_* then, DSC is simply overlap ratio of these two volumes and computed as

$$DSC\left(U_{1},U_{2}\right)=2\frac{\left|U_{1}\cap U_{2}\right|}{\left|U_{1}\right|+\left|U_{2}\right|}=\frac{2.TPVF}{\left(FPVF+TPVF\right)+(TPVF+FNVF)}$$

Specifically, overlap of two segmentations $(U_{1}\cap U_{2})$ indicates true positive volume fraction (TPVF) of the segmentation performance, with respect to both sensitivity and specificity parameters—false negative volume fraction (FNVF) and false positive volume fraction (FPVF). A value of DSC=100 (in percent) indicates a perfect agreement, while a value of 0 indicates no overlap observed [26]. HD, on the other hand, is a metric measuring how boundaries of two segmented objects are in agreement. HD is defined based on how close/far the segmented boundaries are from each other:

$$HD\left(X,Y\right)=\max\left\{sup_{x\in X}inf_{y\in Y} d\left(x,y\right), sup_{y\in Y}inf_{x\in X} d\left(x,y\right)\right\}$$

where *d* is Euclidean distance between points *x* and *y*, sup and inf represent supremum and infimum, and *X* and *Y* indicate boundaries of segmented object and ground truth computed from boundary operation over volumes (i.e., $HD\left(X,Y\right)=HD\left(\partial U_{1}=X,\partial U_{2}=Y\right), \text{ }\partial$ represents boundary). An incremental algorithm, least trimmed square (LTS), and averaging over max-min optimization based computation of HD can be applied instead of conventional max-min optimization of point sets to remove “possible outliers” from HD computation. In our computations, we used average of the single point distances to compute HD in a more robust fashion.

Reproducibility of Random Walk Segmentation and Robustness of Detection Algorithm: We tested reproducibility of the random walk segmentation study based on two experiments. In first experiment, sensitivity of random walk segmentation was assessed by user-defined seeds. All forty images, baseline and follow-up, were used in the segmentation procedure so that each manual seeding procedure was repeated 10 times by putting the seeds randomly over the image regions, while keeping the seeds belonging to the object and the foreground. Resultant segmentations were compared to the ground truth object definition via DSC. The mean, standard deviation, max, and min scores are reported in Table S1. Note that the proposed segmentation algorithm with manual seeding procedure achieved a variation in segmentation accuracy of <1%.

Validation of the Segmentation Algorithm via Phantom Study: We re-iterate our previously shown validation study [22] here. We used an IEC image quality phantom [22], containing six different spherical lesions of 10, 13, 17, 22, 28, and 37 mm in diameter, as illustrated in Figure S1. Two different voxel sizes (2x2x2 and 4x4x4 mm^3^) and two different signal-to-background ratios were considered as reconstruction parameters; different reconstruction parameters are leading to a different resolution, as shown in Figure S1-(a-d). The ground truth of the phantom was simulated from CT and is shown in Figure S1-(e). Variations in resultant segmentation of the spherical lesions in phantoms—due to the use of different reconstruction parameters—are shown in Table S2 below. Note that variations are computed with respect to the ground truth simulations from CT. Automated detection of the lesions from phantoms lead to the same segmentation accuracies, as different localizations of the seeds, within the same foreground, does not change the segmentation accuracies.

Comparison to Threshold-based methods: Fuzzy C-Means (FCM), fuzzy locally adaptive Bayesian (FLAB) and random walk segmentation methods are used for comparison. It has been found that the accuracy of the random walk segmentation algorithm is superior to FLAB and FCM, and FLAB performs better than FCM on the phantom data sets. FLAB and FCM algorithms have average delineation errors spanning from 5-15% and 15-20%, respectively. On the other hand, the random walk segmentation algorithm has the average delineation error over the phantom data as 3.3-16.4%—which is significantly different and better than both methods. This ratio is even smaller (i.e., less than 10%) if objects with a size smaller than 2 cm are not considered in the evaluation. Similarly, FLAB and FCM have overlap ratios (DSC) of 92.17 +/- 4.16 % and 87.31 +/- 5.16, respectively, with respect to the random walk segmentation, where differences between segmentation methods were statistically significant (p<0.01 via t-test). It is also important to note that FCM and FLAB algorithms may need removal of any false positive regions manually. FLAB has reproducibility variation of around 4%, whereas random walk segmentation has a variation of less than 1%.

**
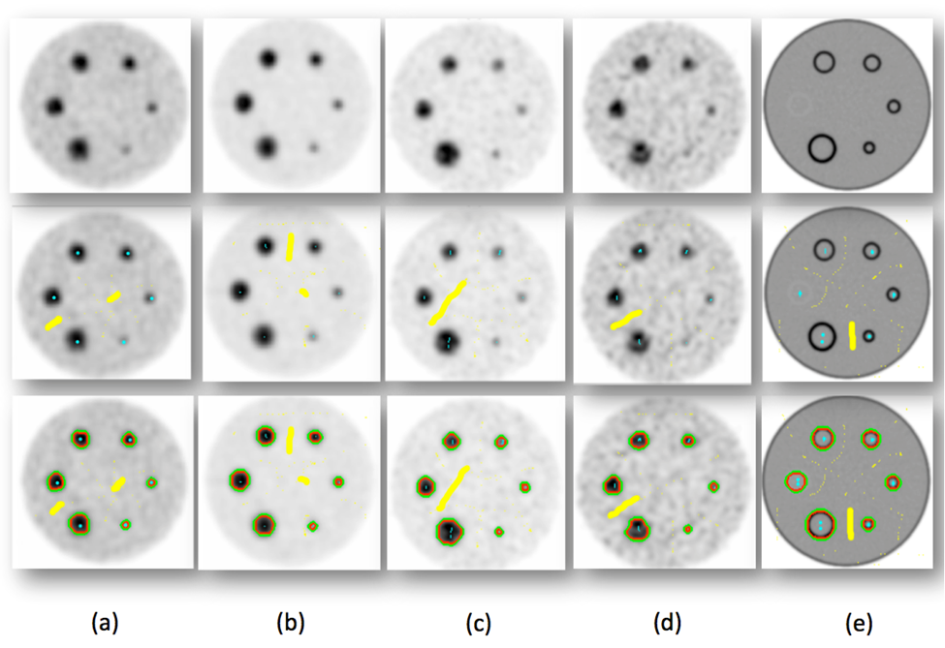
**

**Figure S1:** Phantom images (first row), user labeled foreground and background seeds on phantom images (second row), and random walk segmentation of phantom images based on the seeds (third row) are shown. Phantom images are having the following properties: (a) signal to background ratio 4:1, 64 mm^3^, (b) signal to background ratio 8:1, 64 mm^3^, (c) signal to background ratio 8:1, 8 mm^3^, (d) signal to background ratio 4:1, 8 mm^3^, (e) CT acquisition-ground truth.

**TABLES**

**Table S1:** Reproducibility of random walk segmentation through DSC ratios.

|  | **Mean** | **Std** | **Max** | **Min** |
| --- | --- | --- | --- | --- |
| **DSC (%)** | 99.10 | 0.70 | 99.91 | 96.59 |

**Table S2:** Segmentation accuracy of phantoms, with respect to the ground truth is reported.

|  | **Phantom-a** | **Phantom-b** | **Phantom-c** | **Phantom-d** |
| --- | --- | --- | --- | --- |
| **Mean DSC (%)** | 92.7 | 96.7 | 88.9 | 83.6 |
| **SD of DSC (%)** | 2.99 | 3.18 | 2.62 | 2.68 |
